# Angioneurotic Edema Associated with Haloperidol

**DOI:** 10.1155/2012/725461

**Published:** 2012-09-24

**Authors:** Samrina Kahlon, Cathy Lee, Roger Chirurgi, Getaw Worku Hassen

**Affiliations:** ^1^Metropolitan Hospital Center, New York Medical College, New York, NY 10029, USA; ^2^Department of Emergency Medicine, Metropolitan Hospital Center, New York Medical College, New York, NY 10029, USA

## Abstract

*Background*. Angioneurotic edema is a life-threatening medical emergency that requires urgent diagnosis and treatment. Haloperidol is in the butyrophenone class of antipsychotic medications. Acute anaphylaxis to Haloperidol is very rare and no cases have been reported in literature. *Objective*. To report the association of life-threatening angioneurotic edema with intramuscular Haloperidol. *Case Report*. We present a case of an adult with no known allergies in whom angioneurotic edema with tongue swelling and protrusion developed after the administration of a single IM dose of Haloperidol. *Conclusion*. We propose angioneurotic edema in a rare side effect of Haloperidol. The onset of the symptoms is abrupt, but it may take 12–36 hours to resolve completely. Therefore patient should be monitored for 12–36 hrs.

## 1. Introduction

Haloperidol is an antipsychotic drug in the butyrophenone class. It is used frequently to treat schizophrenia and in the Emergency Department to treat delirium and acute psychosis. Its known common adverse effects include extrapyramidal reactions, restlessness, neuroleptic malignant syndrome, dry mouth, tremor, and weight gain. Angioneurotic edema is a rare adverse effect of haloperidol that has never been described in the literature in the past. We present a case of an adult with no known allergies in whom angioneurotic edema with tongue swelling and protrusion developed after the administration of a single intramuscular dose of haloperidol. The onset of the symptoms is abrupt, but it may take 12–36 hours for symptoms to resolve completely due to the half-life of the drug. We therefore recommend patients to be monitored up to 36 hours after the onset of symptoms.

## 2. Case Report

A 29-year-old male presented to the Emergency Department with a chief complaint of tongue swelling for four hours. The patient reported that he had a similar episode the day before after being given Haloperidol intramuscularly by his psychiatrist. At that time, the patient had received fifty milligrams of Benadryl by his doctor and his swelling resolved. He stated that his tongue swelled up again the next morning without taking any further doses of Haloperidol. There is no history of fever, chills, skin rash, hives, dizziness, upper respiratory symptoms, or trauma. The only significant past medical history is schizophrenia. He has been stable on oral Zyprexa and Risperidone. His medical history was otherwise unremarkable. On physical examination, the patient was mildly anxious, drooling, and speaking with a muffled voice. His entire tongue and uvula were edematous. He had limited movement of the tongue because of the swelling. His vital signs were heart rate of 100, respiratory rate of 18, blood pressure of 151/92, pulse oximetry of 100% on room air, and an oral temperature of 36.7°C (98.0 F). He was alert and oriented, cooperative and in mild discomfort. The patient's neck was supple, with no swelling, muscle spasm or stridor. His lungs were clear bilaterally without wheezing, rales, or rhonchi. The remainder of the physical examination was normal. The patient was given diphenhydramine and epinephrine subcutaneously. The patient improved within minutes as his tongue swelling decreased. He appeared less anxious and his voice quality improved. His angioedema continued to resolve and he was admitted to the medical services for further observation.

## 3. Discussion 

Angioneurotic edema, also known as Quincke's disease and angioedema, is a well-localized edematous condition that variably can involve the deeper skin layers and subcutaneous tissues, as well as the mucosal surfaces of the upper respiratory and gastrointestinal tracts [1–3]. It usually involves the lips, eyelids, genitalia, tongue, or the back of the hands and feet. It can manifest abruptly, compromising the patient's airway within minutes, and may take hours to days to resolve.

Haloperidol is a commonly used antipsychotic medication. It is a butyrophenone derivative and resembles droperidol derivatives pharmacologically [4]. Four cases of angioneurotic edema have been reported after droperidol administration [5]. Review of the literature revealed no previous report of angioneurotic edema after the administration of Haloperidol. This patient, with no history of any allergies to medications, suddenly developed angioedema after he received intramuscular Haloperidol. The patient was given 50 mg diphenhydramine and the symptoms resolved but the patient had similar symptoms when he woke up next morning without taking the medication. Repeat doses of diphenhydramine and epinephrine were required before his symptoms subsided in the Emergency Department. 

Angioneurotic edema is potentially a life-threatening condition with a variety of causes. It has been described by three different pathophysiologic pathways: IgE-mediated immediate hypersensitvity reactions, complement-mediated reactions, and specific drug reactions [6]. Our patient experienced an immediate hypersensitivity reaction to a specific antigen. Medications do not react as antigens unless bound to a carrier protein, as they are of low molecular weight. The drugs metabolite combining with the proteins that form the antigen is responsible for starting the immune response [7].

Our patient was given intramuscular Haloperidol by his psychiatrist. Plasma-levels reach their maximum within 20 minutes after injection and the patient developed angioneurotic edema. We present a case of angioedema following Haloperidol administration in a previously healthy male with no history of drug allergies. Physicians should also remember that symptoms may resolve immediately after the administration of epinephrine and diphenhydramine but they should monitor the patient for at least 36 hrs before they discharge the patient as the angioedema may not have resolved completely or may reoccur as the half life of Haloperidol is 12–36 hrs.
